# Construction Notes

**Published:** 1893-08-19

**Authors:** 


### CONSTRUCTION NOTES.

The Parkwood Convalescent Home, Swanley.—Mr. Peter Reid, of Norfolk Street, Park Lane, has provided the munificent sum of £100,000 for the purpose of establishing this convalescent home, which is to be in connection with the London, Guy's, St. Thomas's, the Middlesex, Westminster, and St. Mary's hospitals. Mr. Reid's philanthropic scheme has since been considerably enlarged through the unsolicited and purely spontaneous generosity of some of the founder's personal friends, who added the sum of £50 000 to the primary fund. The freehold of an estate of 50 acres, known as Parkwood, in the Swanley district, has been secured, on which ground the present building has been erected from plans prepared by Mr. Edward B. I'Anson, of Laurence Pountney Hill. The home provides accommodation for 120 patients—80 for male and 40 for females. The work of erecting the building was carried out by Messrs. Shillitoe and Son, builders, of Bury St. Edmunds. The building faces due south, and is approached by a wide and lengthy drive. The style of the building is domestic. It is three storeys high, and has a total frontage of 313 feet; it is faced with red brick and stone dressings, the upper part of the walls being covered with red hanging tiles, and the roof is covered with Broseley tiles. The central or administrative block provides accommodation for the meetings of the trustees, and for the medical officer, the matron, assistant matron, nurses, and domestic staff. To the west of this block is the men's wing, consisting on the ground floor of four day rooms and two smoking rooms, with verandahs. The east wing is appropriated to women, with three commodious rooms on the ground floor, and verandahs. To the north of the central block is the large dining hall, which has separate corridors leading from the men's and women's wings, and which is capable of seating 200 persons. Behind this dining hall are the kitchens, nurses' dining room, and domestic offices, which are only one storey high. There are separate staircases for the administrative block, and for each of the patients' wings, and on the upper floors, bed-room accommodation is provided, together with lavatories and baths. Detached from the building on its north side is a spacious and liberally fitted-up laundry. In the rear of the main building, and connected with it by a handsome corridor, is a chapel, which has been provided by the generosity of Mr. Ebenezer Homan, of Finchley, the erection and completion of which cost upwards of £5,000. In addition to this gift, Mr. Homan has contributed £3,000 towards the endowment of a chaplaincy fund, and has also presented a large number of pictures and books for the use of the inmates of the home. In the construction of the home every sanitary requirement has, throughout, had careful attention, and the interior arrangements are replete with all that can add to the comfort of the inmates. The cost of the structure, irrespective of the purchase of the freehold, is considerably over £40,000, and after defraying the cost of the site and all expenses attending the erection and furnishing of the building, the trustees will have an income from the endowment fund of £3,500 towards the maintenance of the institution, which, as far as possible, will be conducted on a self-supporting basis. It is an important feature of the home that not only convalescents proper, but also hospital patients who are still in need of medical or surgical treatment or nursing will be allowed to participate in the benefits of the institution, and the *personnel* of the establishment comprises a staff of fully-trained nurses, under the control of the matron, and the medical officer, Dr. Hugh Smith, of Farningham.

